# Paying Patients in Bermondsey Infirmary

**Published:** 1921-03-26

**Authors:** 


### Paying Patients in Bermondsey Infirmary.

Bermondsey Board of Guardians have decided to set aside in their maternity wards at their infirmary twelve beds for maternity cases, at a minimum charge of £1 1s. a week. This is a great need in Bermondsey, with its overcrowded population, cramped together in congested rooms, where there is no chance of the mother having the nursing and medical attention requisite. Other London Boards of Guardians have been urged by those engaged amongst the poor to provide the same facilities for paying patients, but they have had to let any possible scheme slide because of the present condition of the Poor Law that only destitute persons can be admitted. The lack of the nursing and medical attention in a home comes within the definition of destitution as laid down by the Local Government Board many years ago. Dr. Addison, in a letter addressed to the Conference of Poor Law Associations held in London on February 17 last, with regard to utilising vacant beds in Poor-law infirmaries for paying patients, stated that the Guardians had no legal power to make such provision, and that if this was contemplated legislative powers would first have to be obtained from Parliament; but he added that the Ministry of Health would raise no objection to individual non-destitute cases being admitted to an infirmary. For some years this has been done in many London infirmaries.

